# Supplementary material for: Empirical methods for controlling false positives and estimating confidence in ChIP-Seq peaks
Source: BMC Bioinformatics. 2008 Dec 5;9:523. doi: 10.1186/1471-2105-9-523 (PMC2628906; doi:10.1186/1471-2105-9-523)
Supplement: Additional File 1 — A variety of html documents from the USeq web site detailing the available applications, their best usage, output file type descriptions, command line menus, etc. [file 1471-2105-9-523-S1.zip › USeqUserGuides/JavaDoc.zip!/JavaDoc/trans/roc/Bin.html]

Bin


|  |  |  |
| --- | --- | --- |
| **Overview** \| **Package** \| **Class** \| **Use** \| **Tree** \| **Deprecated** \| **Index** \| **Help** | |  |
| PREV CLASS   **NEXT CLASS** | **FRAMES**    **NO FRAMES**     **All Classes** |  |
| SUMMARY: NESTED \| FIELD \| CONSTR \| METHOD | DETAIL: FIELD \| CONSTR \| METHOD |  |


---


## trans.roc Class Bin

```
java.lang.Object
  trans.roc.Bin
```

---

``` public class Bin extends java.lang.Object ```

Score collector for Sgr apps.

---

**Constructor Summary**

`Bin()`

`Bin(double start,
double stop)`


| **Method Summary** | |
| --- | --- |
| `boolean` | `contains(double score)` |
| `double` | `getFdr()` |
| `int` | `getNumNegatives()` |
| `int` | `getNumPositives()` |
| `double` | `getStart()` |
| `double` | `getStop()` |
| `void` | `incrementNegatives()` |
| `void` | `incrementPositives()` |
| `void` | `setFdr(double fdr)` |
| `void` | `setNumNegatives(int numNegatives)` |
| `void` | `setNumPositives(int numPositives)` |
| `void` | `setStart(double start)` |
| `void` | `setStop(double stop)` |
| `java.lang.String` | `toString()` |

**Methods inherited from class java.lang.Object**

`equals, getClass, hashCode, notify, notifyAll, wait, wait, wait`

**Constructor Detail**

### Bin

```
public Bin()
```

---


### Bin

```
public Bin(double start,
           double stop)
```


**Method Detail**

### contains

```
public boolean contains(double score)
```

---


### incrementPositives

```
public void incrementPositives()
```

---


### incrementNegatives

```
public void incrementNegatives()
```

---


### toString

```
public java.lang.String toString()
```

:   **Overrides:**: `toString` in class `java.lang.Object`

---


### getNumNegatives

```
public int getNumNegatives()
```

---


### setNumNegatives

```
public void setNumNegatives(int numNegatives)
```

---


### getNumPositives

```
public int getNumPositives()
```

---


### setNumPositives

```
public void setNumPositives(int numPositives)
```

---


### getStart

```
public double getStart()
```

---


### setStart

```
public void setStart(double start)
```

---


### getStop

```
public double getStop()
```

---


### setStop

```
public void setStop(double stop)
```

---


### getFdr

```
public double getFdr()
```

---


### setFdr

```
public void setFdr(double fdr)
```


---


|  |  |  |
| --- | --- | --- |
| **Overview** \| **Package** \| **Class** \| **Use** \| **Tree** \| **Deprecated** \| **Index** \| **Help** | |  |
| PREV CLASS   **NEXT CLASS** | **FRAMES**    **NO FRAMES**     **All Classes** |  |
| SUMMARY: NESTED \| FIELD \| CONSTR \| METHOD | DETAIL: FIELD \| CONSTR \| METHOD |  |


---
